# A new lesser metatarsophalangeal joint replacement arthroplasty design - in vitro and cadaver studies

**DOI:** 10.1186/s12891-021-04257-x

**Published:** 2021-05-07

**Authors:** Nikiforos P. Saragas, Paulo N. F. Ferrao, Andrew Strydom

**Affiliations:** 1 The Orthopaedic Foot and Ankle Unit, Suite 303 Netcare Linksfield Hospital, 24 12th Avenue, Linksfield West, Johannesburg, 2192 South Africa; 2 Foot and Ankle Unit, Division of Orthopaedic Surgery, University of the Witwatersrand, Johannesburg, South Africa; 3 Netcare Sunninghill Hospital, Suite 3A, -2 Level, Westwing, Cnr Nanyuki & Witkoppen Road, Sunninghill, Johannesburg, 2157 South Africa

**Keywords:** Lesser metatarsophalangeal joint, Replacement arthroplasty, New design, Freiberg’s infraction, Arthritis, Cadaver studies

## Abstract

**Background:**

Isolated degenerative joint disease and/or Freiberg’s infraction of the lesser metatarsophalangeal joint, although not frequent may become debilitating in the younger individual. Currently, once conservative management fails, the mainstay of treatment is debridement and excision-interposition arthroplasty. Replacement arthroplasty has been ineffective in the long term as the joints are subject to severe repetitive fatigue loading over small articulating surfaces through a wide range of motion. This is an in vitro and cadaver study of a new design replacement arthroplasty developed by the senior author.

The aim of this study is to evaluate this novel replacement arthroplasty of the lesser metatarsophalangeal joint in a laboratory setting and cadaver implantation.

**Methods:**

This three-component mobile bearing device is made of titanium and high density polyethylene which evolved over 4 years. It was subjected to 5,000,000 cycles in a laboratory under physiological and excessive forces to assess resistance to fatigue failure and wear pattern of the polyethylene liner. Following these tests, it was implanted in 15 fresh frozen cadavers at various stages of its development, during which the surgical technique was perfected. Range of motion and stability was tested using custom made instrumentation in four cadavers. The implant was inserted in a further two cadavers by an independent foot and ankle surgeon to check reproducibility.

**Results:**

The device showed almost no signs of wear or surface deformation under physiological forces. The surgical technique was found to be simple and reproducible in the cadaver trial.

The average dorsiflexion was 28.5° and 28.9° pre- and post-implant respectively. The average plantar flexion was 33.8° and 20.8° pre- and post- implant respectively. The joints were stable both pre- and post-operatively. Post-operative stability was objectively assessed for dorsal displacement and dorsiflexion using a 5 kgf (49 N) and was found to be excellent.

**Conclusion:**

This novel lesser metatarsophalangeal joint replacement arthroplasty has been developed as an option in the surgical treatment of symptomatic degenerative joint disease and/or Freiberg’s infraction resistant to conservative treatment. The implant was found to be durable and resistant to wear in the laboratory testing. The cadaver studies have shown it to require minimal specialized instrumentation with good surgical reproducibility.

This proof of concept study is the basis for clinical trials.

## Introduction

Degenerative arthritis of the lesser metatarsophalangeal joint (LMTPJ) in the foot is a relatively uncommon condition as compared to the inflammatory arthritides. Often the arthritis is isolated to one joint and commonly due to previous trauma or Freiberg’s infraction. This condition may become debilitating in the younger individual. There is a paucity of published information on alternative treatment options as far as arthroplasties are concerned when conservative therapies fail. Excisional and interpositional arthroplasties using various tissues have and are still being used as the main surgical treatment option [1–15].

Joint replacement arthroplasty has been used in the end stages of the disease [16]. Silicone [17–26], metal [17–29] and ceramic [30] LMTPJ replacement arthroplasty as well as osteochondral autograft transplantation [6] have been reported with mixed success.

Currently there is no effective replacement available. These joints are subject to severe repetitive fatigue loading over small articulating surfaces through a wide range of motion.

Most published series stem from the 1970’s and 1980’s [17–25]. The initial simple silicone spacers and silicone ball [26] without stems were improved by the addition of prongs to increase stability. The Swanson prosthesis, originally designed for the hand, has been used for the LMTPJs [17–25]. The Nicolle, the Calnam-Nicolle and the Niebauer-Cutter hinged prosthesis had been adapted for LMTPJ arthroplasty. The reported cases are too few and short term to make recommendations for their use [18, 24].

In a series by Cracchiolo, 31 s MTPJs in 28 patients were replaced by a double-stem silicone implant and a single-stem in one. Six of these patients had Freiberg’s infraction. Severe subluxation or dislocation of the 2nd MTPJ was present in 26 of 32 ft. None of the Freiberg’s infraction group had significant deformity. At an average follow-up of 37 months, a good subjective result was recorded in 63% and good with reservations in 25%. Transfer metatarsalgia was a reported complication in both the reservation and failure groups [17].

Sgarlato has advocated the use of a double-stem silicone prosthetic implant in several difficult-to-treat conditions. He found it to be successful in older (over age 50 years) patients [22].

In a small cohort of patients with a short follow-up, Townshend and Greiss used a total ceramic arthroplasty for painful destructive disorders. Eight of nine patients reported good or excellent results at a mean follow-up of 23 months [30].

All results quoted were from studies with a small number of patients to make any strong argument for favoring any of the procedures described meaningless.

The objective of this study is to design an effective replacement arthroplasty of the second and third metatarsophalangeal joint for end stage arthritis or symptomatic Freiberg’s infraction to add to the armamentarium of the foot and ankle surgeon. The authors hypothesize that the design of this implant will effectively simulate the lesser metatarsophalangeal joint in both laboratory setting and cadaver trials so as to follow with clinical trials.

## Materials and methods

**Fig. 1:**
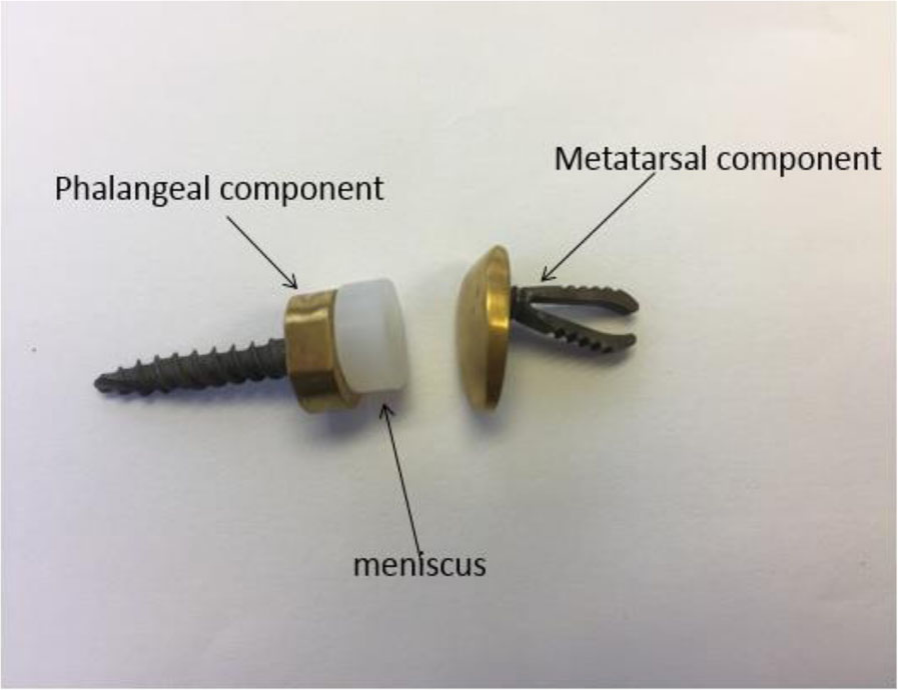
Lesser metatarsophalangeal joint implants

A novel implant was designed and developed by the senior author (NPS) (Fig. 1 - Lesser metatarsophalangeal joint implants).

The implant is considered to be more of a resurfacing rather than a metatarsal head replacement so as not to interfere with the plantar condyles of the metatarsal head.

The implant can be visualized as a device permitting a stable yet mobile bearing unit.

The design was conceptualized as being low constrained with a high conformity that provides axial rotation and allowing more sagittal than coronal motion.

This new lesser metatarsophalangeal joint replacement is based on a three-component implant.

Although it is considered to be a three-component implant, the mobile bearing meniscus clips onto the phalangeal component via a peg which is smaller in diameter from the corresponding phalangeal socket allowing for multidirectional gliding at this interface, thus providing both stability and decreasing the torsional forces. The mobile bearing can rotate 360°.

The metallic components are made of titanium with a grid blasted under surface for maximum bony ingrowth. The articular surface has a titanium nitride finish for hardness. The meniscus is made of high-density polyethylene.

The implant allows for plantar and dorsiflexion with an element of medio-lateral translation and axial rotation. An integral part of the procedure is the soft tissue balancing of the joint. The plantar condyle is preserved with this implant so that the weight bearing status of the involved metatarsal will be maintained thus avoiding transfer lesions. The implant may be used as a total or hemi arthroplasty. The fixation is intramedullary (a novel “spring” fixation system for the metatarsal and a screw fixation for the phalangeal component) and the implant has high conformity and low constraint to withstand the stresses applied on it by walking and weight bearing, thus minimizing wear.

A number of implants of various sizes were manufactured for the purpose of the study. The sizes were determined by accurate skeletal measurements of the metatarsal heads and base of the proximal phalanges by using digital callipers. Forty adult skeleton feet were used as per the statistician’s advice.

Female and male skeletons were included randomly from the anatomy department. The authors were concerned in creating a range of sizes that would accommodate both genders.

Radiographs of living subjects were used to measure the medullary diameter of the metatarsal and proximal phalanx. The x-rays adhered to the international standard weight bearing protocol of foot x-rays.

The combination of these parameters allowed the researchers to have a range of implant sizes manufactured for implanting into the cadavers. It was found that by using two phalangeal screw sizes, 98% of the adult population was accommodated.

### Cyclical testing in the laboratory

**Fig. 2:**
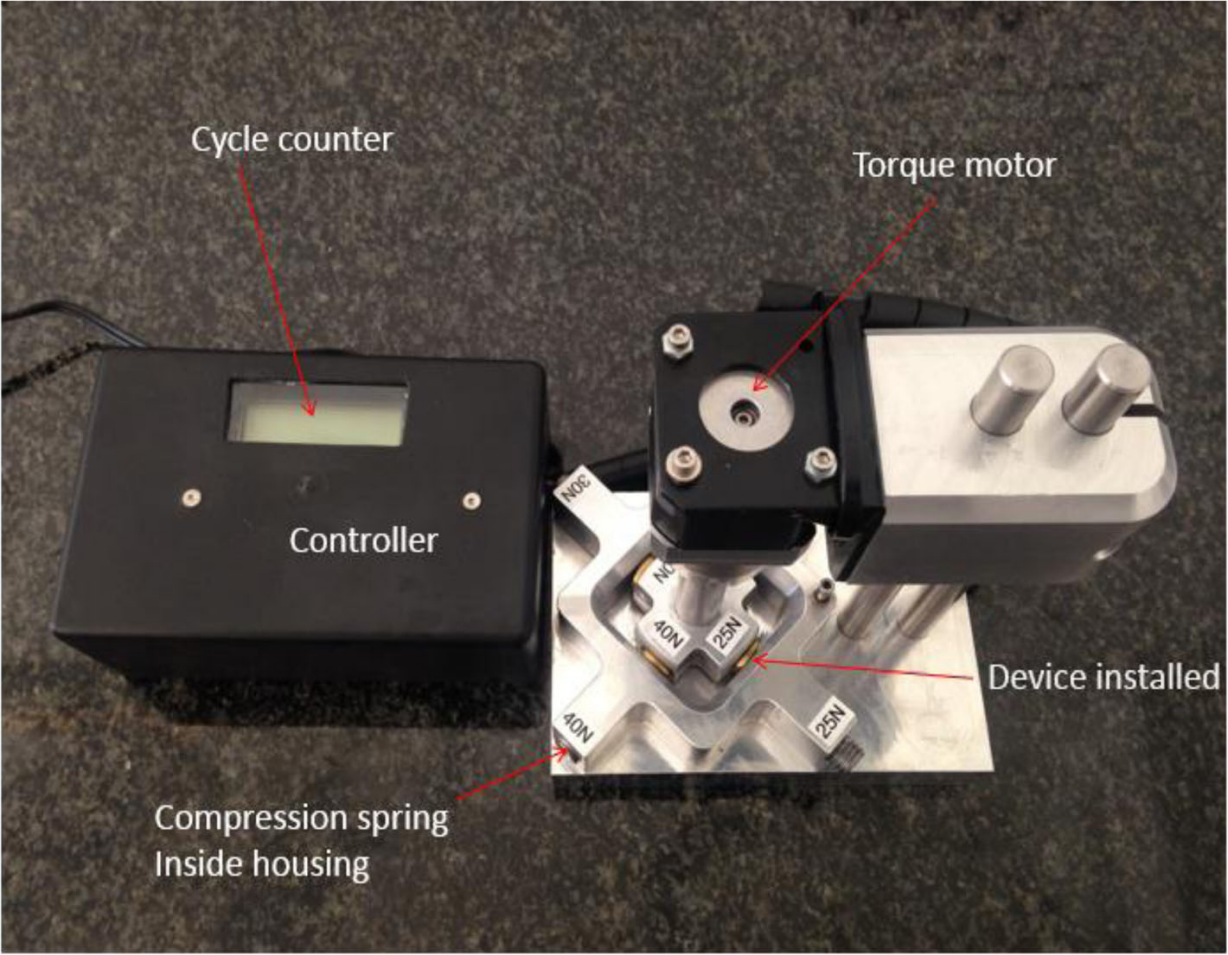
Cyclical testing instrumentation – four stations

For the purpose of testing this medical implant, a mechanical test apparatus was designed in conjunction with bioengineers and manufactured to simulate the articulation of the LMTPJ in the human foot. The apparatus incorporates four stations for testing four implants at different forces simultaneously (Fig. 2 - Cyclical testing instrumentation – four stations). The compressive forces were applied during cyclic articulation by means of cylindrical helical silicone compression springs. The applied compression force was derived from the amount of deflection of the compression springs.

**Fig. 3:**
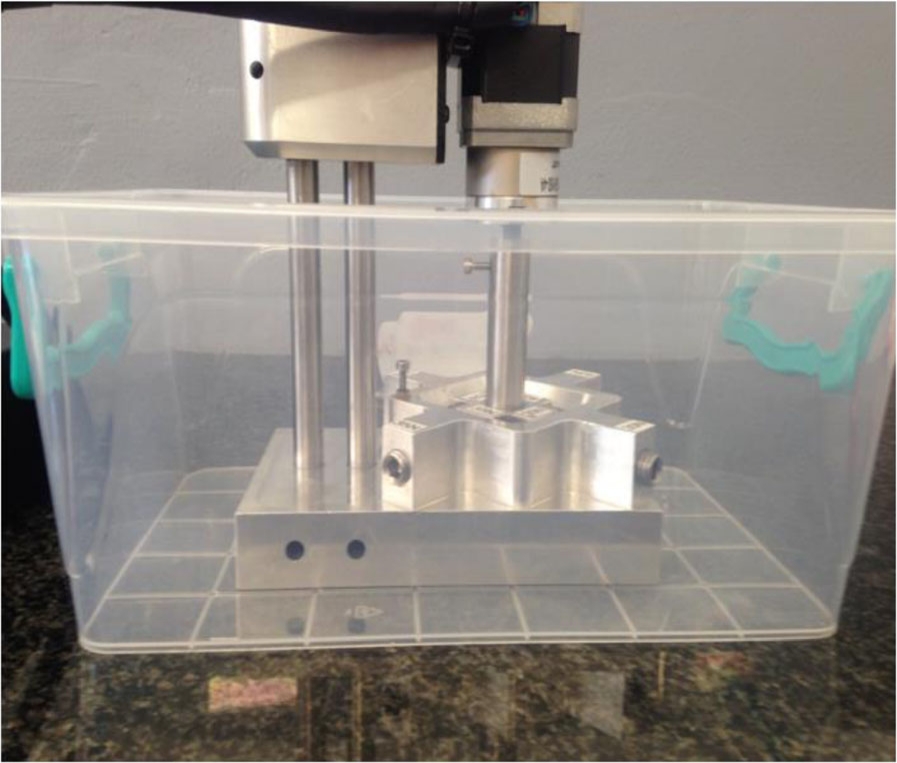
Cyclical testing instrumentation set-up

The articulation was generated using a 12-V DC torsion motor capable of articulating each implant device to a pre-set angle at a frequency of 3 Hz. Testing was conducted in de-ionized water in order to minimize any possible influence by the ions found in normal water. Each test station with its installed implants, was submerged in the de-ionized water (Fig. 3 - Cyclical testing instrumentation set-up). In addition, the test apparatus was covered for the duration of testing to prevent any foreign debris from entering the test environment.

In series one, four implants were tested. These implants were subjected to 5 million cycles each at physiological compressive forces of 3 N, 4 N, 6 N and 8 N respectively [ 31]. The implants were subjected to 5 million cycles, after which wear damage at the contact surface of each implant was captured by means of photographic imaging and thickness measurements of the meniscus. Measurements were carried out with a TESAMASTER Standard High Precision Micrometer with Digital Counter reading down to 1 μ and the water was assessed for polyethylene particles.

In series two, four implants were subjected to excessive compressive forces of 25 N, 30 N, 35 N and 40 N respectively. Once again wear damage at the contact surface of each implant was captured and the water assessed for polyethylene particles.

### Cadaver trials

During the evolution of the implant design, 15 cadavers were used over a period of 4 years. The cadaveric specimens were utilised as part of specialised foot and ankle training workshops run by the authors; the specimens were obtained through standard procurement processes with all necessary permissions.

**Fig. 4:**
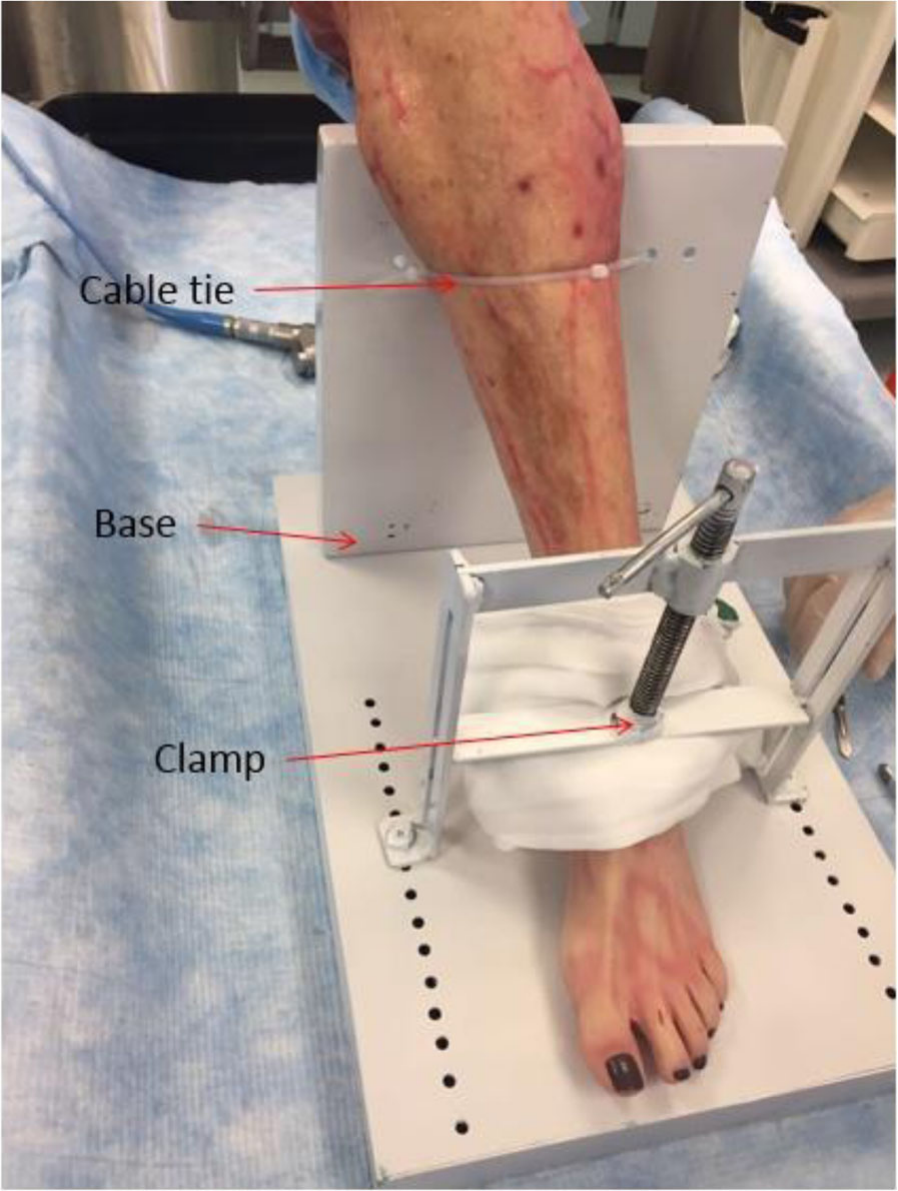
Cadaver testing set up

At the final cadaver trial stage when the final product was tested, four cadavers (four toes) were used. After the fourth specimen, it was noted that all the measurements were remarkably similar and further specimens would prove to be unnecessary and unnecessarily expensive (Fig. 4 - Cadaver testing set up). Only the second metatarsophalangeal joint was tested. There were two male (specimens 1 and 3) and two female (specimens 2 and 4) cadavers.

A further two devices were then implanted in fresh frozen cadavers by an independent foot and ankle surgeon. Radiographs were obtained at this stage.

### Surgical technique

**Fig. 5:**
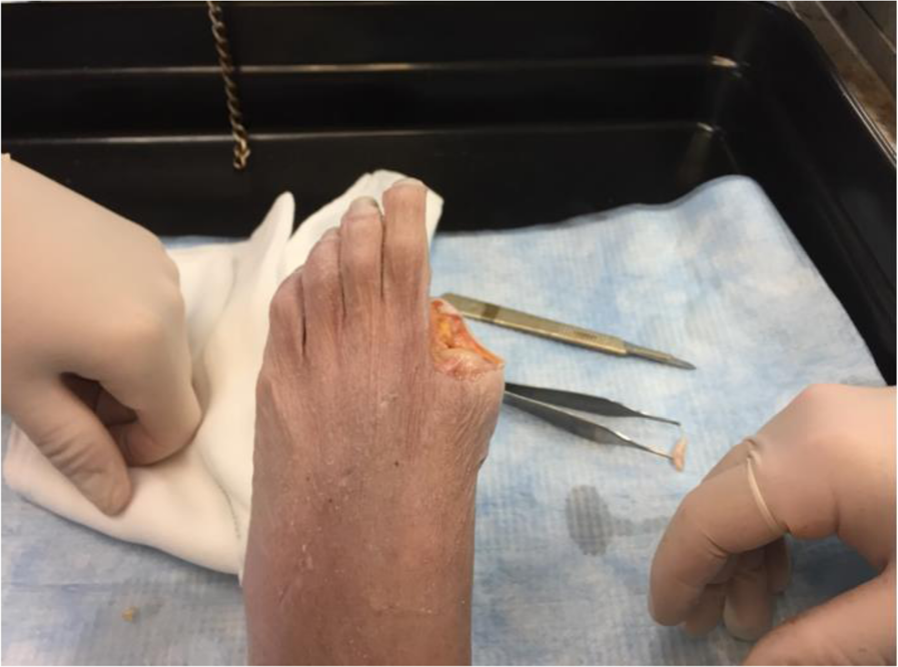
Hallux disarticulation for application of electro-goniometer

The hallux had to be disarticulated at the metatarsophalangeal joint (MTPJ) in order to accurately test the range of motion of the implanted device with a custom-made measuring tool (Fig. 5 Hallux disarticulation for application of electro-goniometer).

A dorsal longitudinal midline incision centred over the lesser metatarsophalangeal joint is made. The incision is deepened between the extensor digitorum longus and extensor digitorum brevis tendons down to capsule. The capsule is split longitudinally.

The collateral ligaments are dissected off the proximal phalanx. Three mm of the base of the proximal phalanx is excised using an oscillating saw and the proximal phalanx is hyper plantar flexed. More bone is excised as required in order to maximize range of motion. The plantar plate is left intact.

**Fig. 6:**
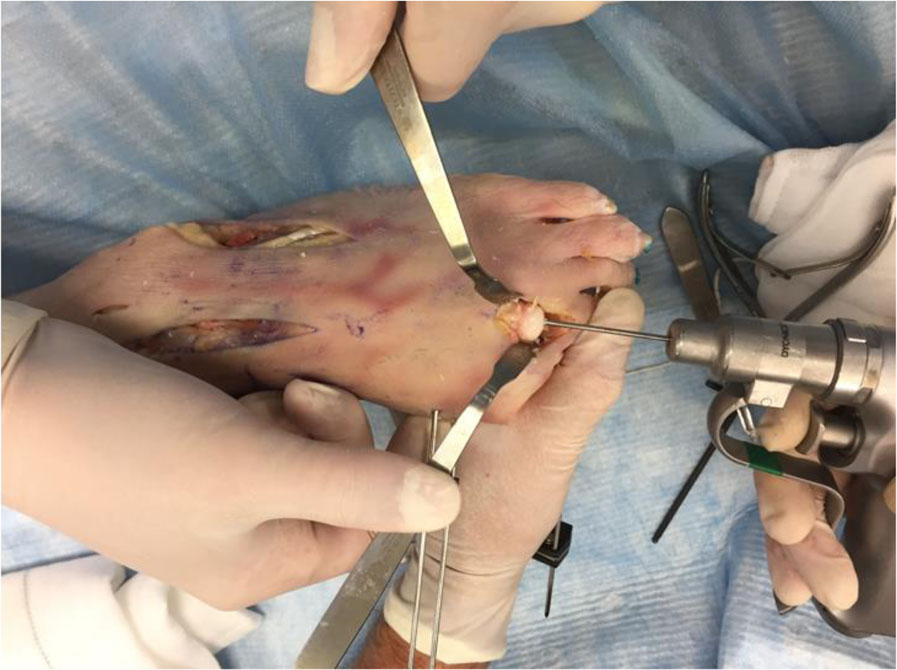
Guide wire place in the metatarsal

**Fig. 7:**
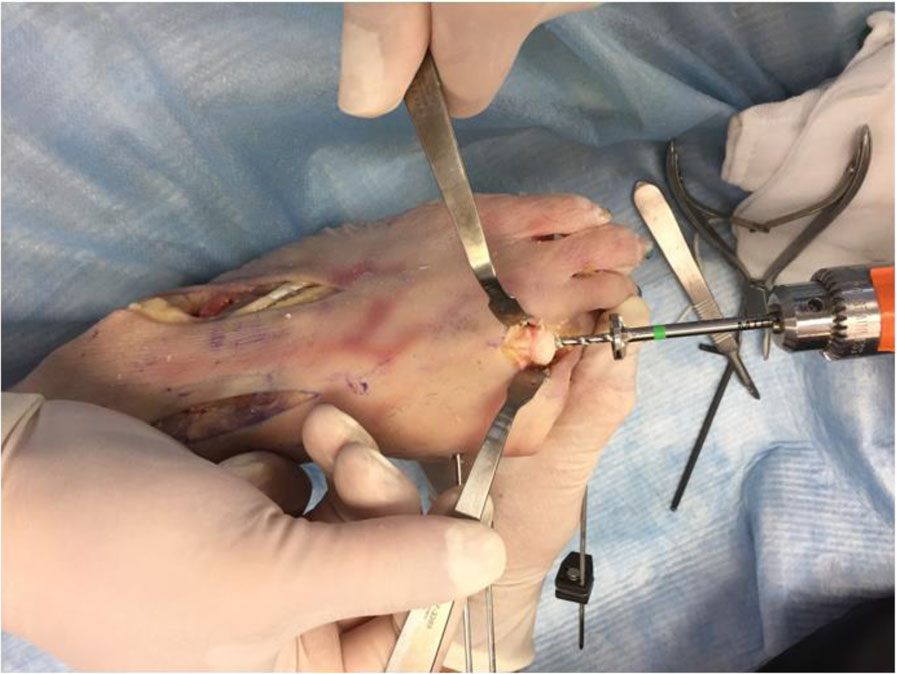
Cannulated reamer over the guide wire

**Fig. 8:**
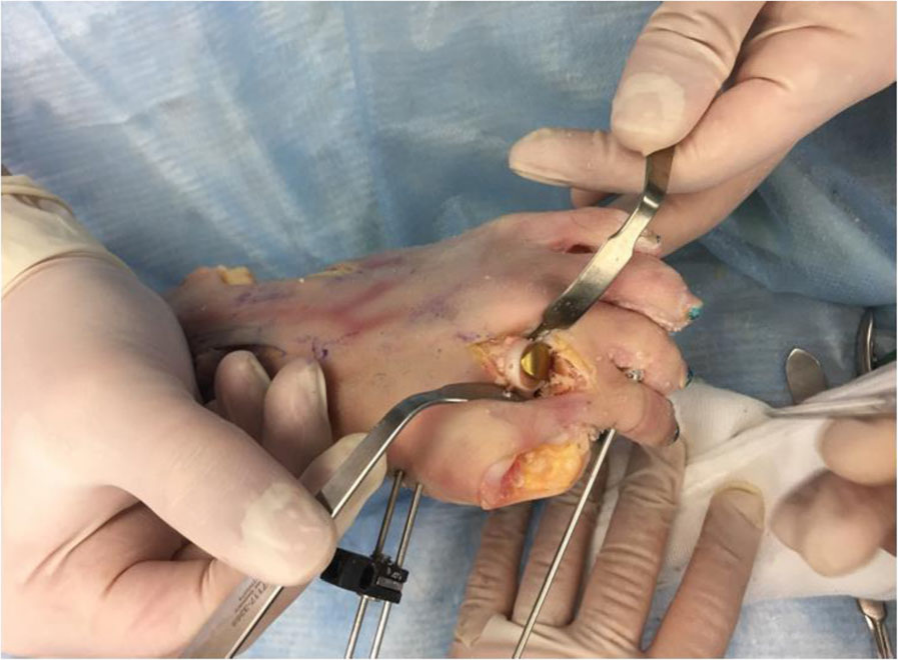
Metatarsal component in place

The edges of the metatarsal head is contoured by debridement of all osteophytes in order to restore a spherical head. A guide wire is driven into the metatarsal head to centralize the component in the shaft (Fig. 6 - Guide wire placement in the metatarsal). A cannulated reamer is then used to prepare the metatarsal head (Fig. 7 - Cannulated reamer over the guide wire). The metatarsal component is then inserted into the metatarsal head and gently impacted in place (Fig. 8 - Metatarsal component in place). The phalangeal component is then screwed into the phalanx.

**Fig. 9:**
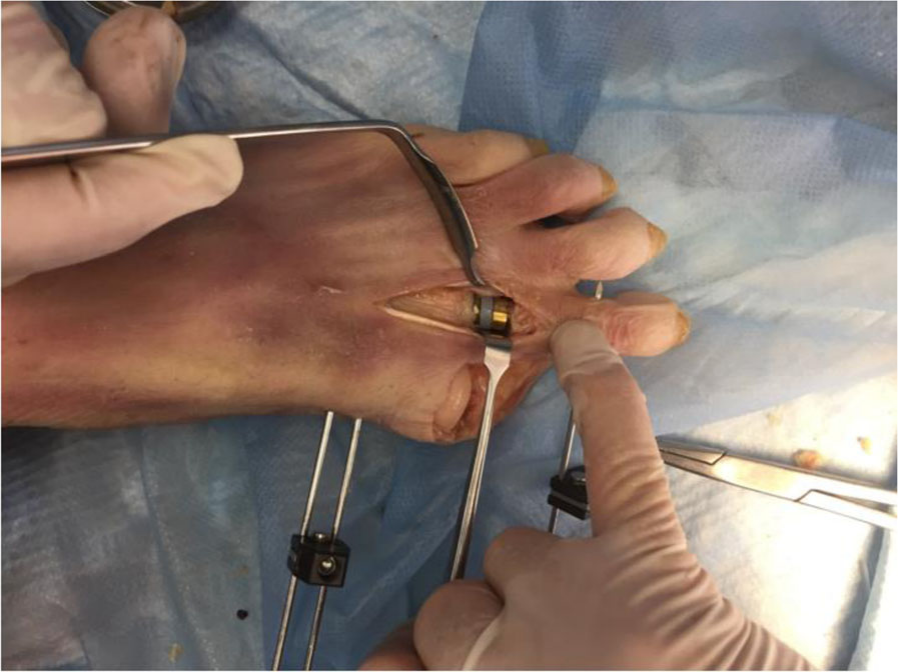
Complete lesser metatarsophalangeal replacement in situ

The trial liners are used to determine the size of the plastic bearing meniscus. Once the soft tissue is well balanced, the correct size polyethylene is inserted into place (Fig. 9 - Complete lesser metatarsophalangeal replacement in situ). Stability and range of motion is checked. The soft tissue is repaired and once again stability, alignment and range of motion are checked.

**Fig. 10:**
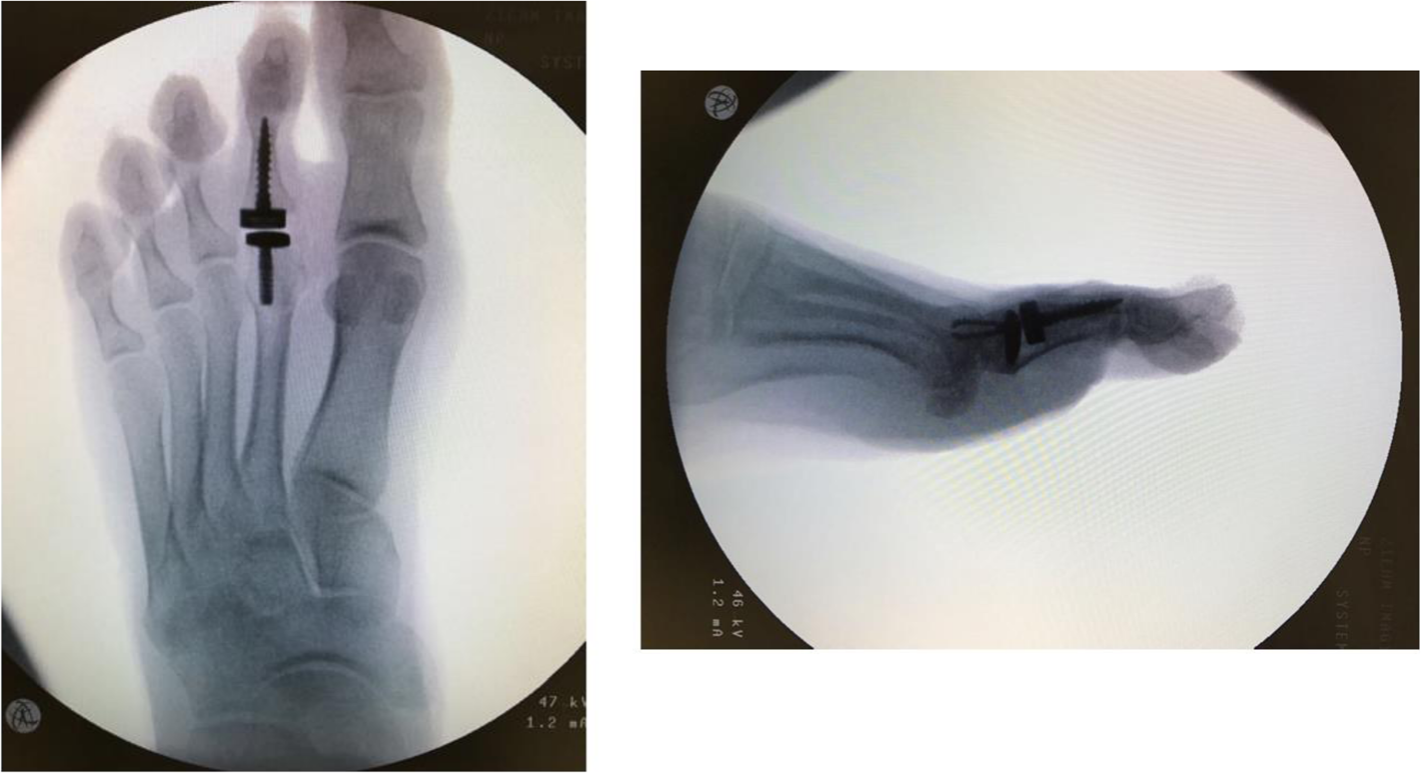
Radiographic appearance of the implant (antero-posterior and lateral views)

X-ray facilities were available for two cadaver specimens (separate from the four cadavers that were tested) to simulate live surgery and obtain radiographs of the implant in the cadaver foot (Fig. 10 - Radiographic appearance of the implant (antero-posterior and lateral views)). No measurements were performed hence there was no need to amputate the hallux.

### Range of motion measurements

**Fig. 11:**
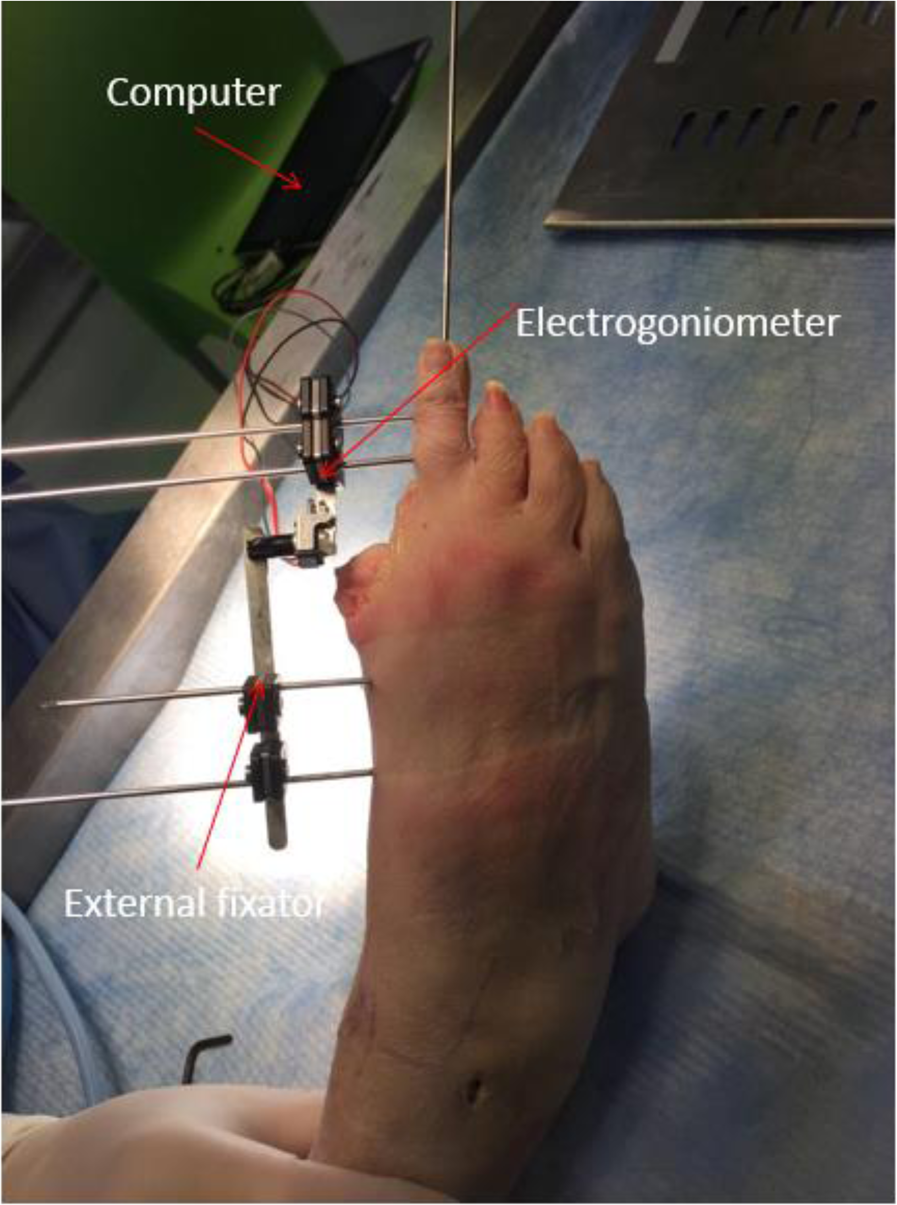
Electro-goniometer for range of motion measurement

A custom-made electro-goniometer and open-source simulation software Ardino Software (IDE) was used (Fig. 11 - Electrogoniometer for range of motion measurement). Range of motion of the pre- and post-implanted LMTPJ was recorded. Prior to the measurement, the LMTPJ was taken through several cycles in order to reach a point of resistance by the same examiner.

### Stability

**Fig. 12:**
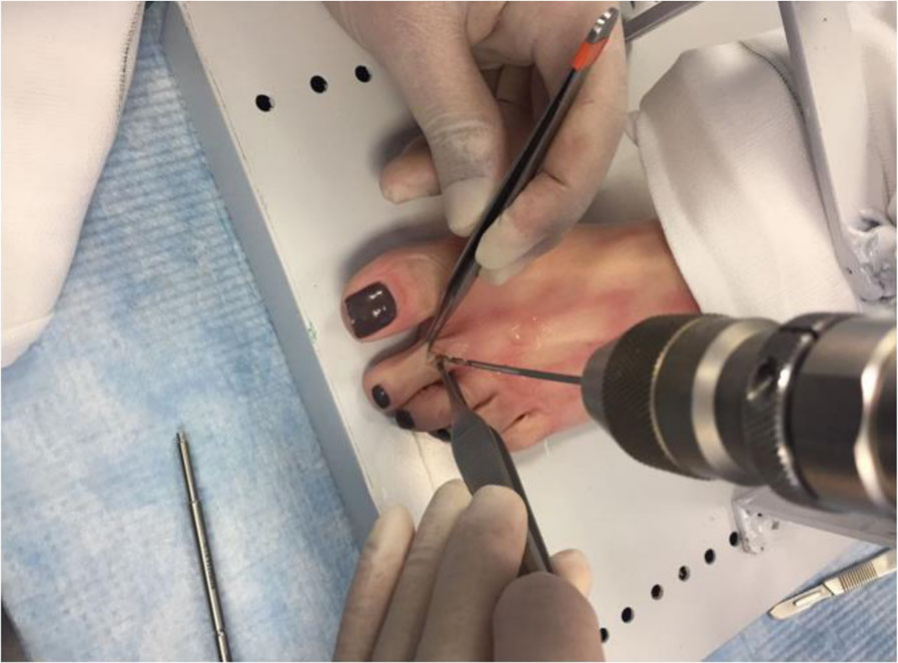
Screw implanted in proximal phalanx for the purpose of stability testing

Stability of the pre- and post-implanted joints was performed clinically with a drawer test as well as using a custom-made device. A screw implanted into the proximal phalanx was used for this purpose (Fig. 12 - Screw implanted in proximal phalanx for the purpose of stability testing). In consultation with the engineers, the friction and friction losses through the set-up were found to be negligible.

**Fig. 13:**
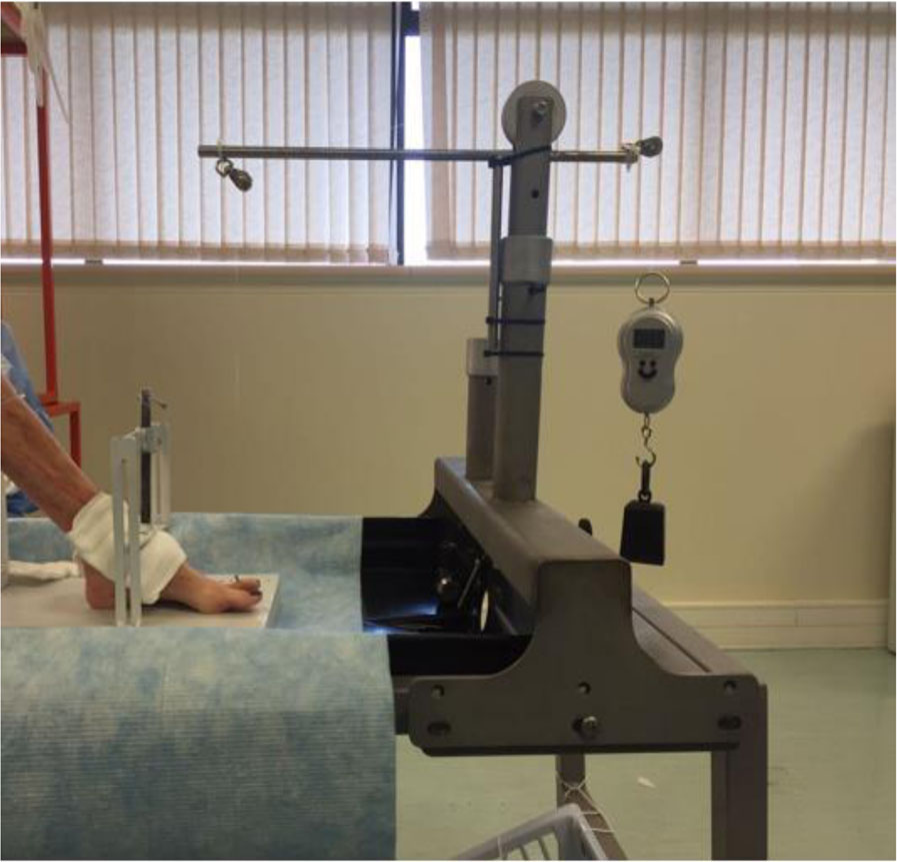
Stability testing setup

A weight force of 5 kg (F = m x a) equalling 49 N was used. This force was shown on previous cadaveric studies to disrupt the soft tissue stabilizing factors of the LMTPJ and is thus seen as very conservative. On average the subluxation stability of the intact joints is around 25 N in a dorsal or superior direction and approximately 16 N in dorsiflexion [32, 33] (Fig. 13 - Stability testing setup).

## Results

### Laboratory results

**Fig. 14:**
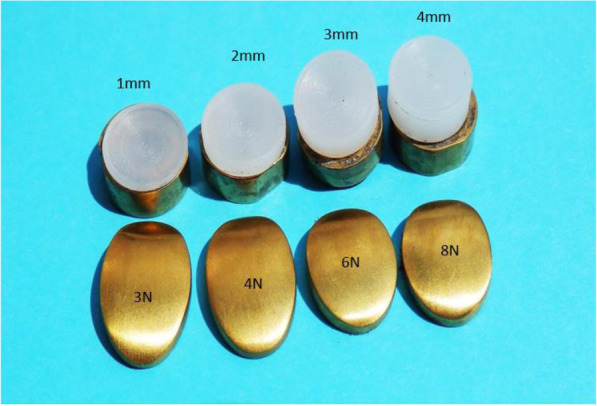
The four implants each with the respective compressive forces as well as the sizes after completing 5,000,000 cycles at physiological forces

From the photographic images captured after testing, it was clear that almost no sign of wear or surface deformation is visible on all four implants tested at the respective physiological compression forces (Fig. 14 - The four implants each with the respective compressive forces as well as the sizes after completing 5,000,000 cycles at physiological forces).

Moreover, the contact surfaces of all four titanium implants show no discolouring after 5 million cycles. Thickness measurements prior and after testing also showed no changes measured in micrometres (Table 1). No polyethylene particles were found in the de-ionized water.

**Table 1:** Measurements pre and post endurance testing after 5,000,000 cycles at physiological forces

Implant size	Original thickness (mm)	Final thickness (mm)
1 × 9 mm - 3 N	1.00	1.00
2 × 9 mm - 4 N	2.00	2.00
3 × 9 mm - 6 N	3.00	3.00
4 × 9 mm - 8 N	4.00	4.00

**Fig. 15:**
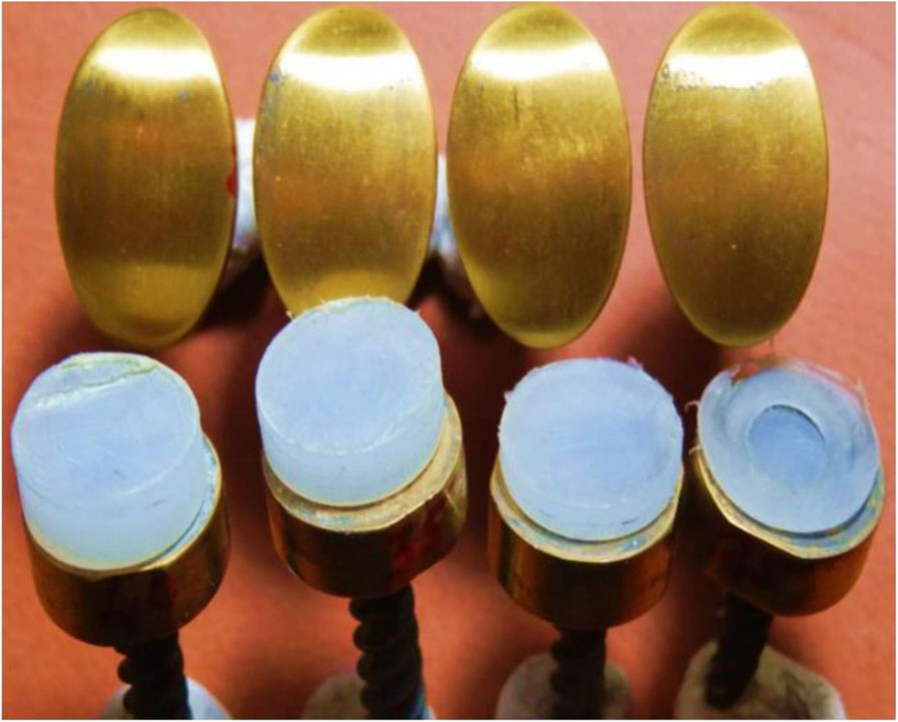
Wear of contact surfaces post testing after 5,000,000 cycles at excessive forces

Significant wear was evident on all four inserts after testing at excessive forces (Fig. 15 - Wear of contact surfaces post testing after 5,000,000 cycles at excessive forces).

Table 2 shows the measurements pre and post endurance testing.

**Table 2:** Measurements pre and post endurance testing after 5,000,000 cycles at excessive forces

Implant size	Original thickness (mm)	Final thickness (mm)
1 × 9 mm - 25 N	1.00	0.45
2 × 9 mm - 30 N	2.00	1.55
3 × 9 mm - 35 N	3.00	2.55
4 × 9 mm - 40 N	4.00	3.56

## Cadaver result

### Range of motion

A total of four dorsiflexion and plantar flexion measurements were included in the study.

The LMTPJ dorsiflexion both pre- and post-implant varied widely from 12° – 52° and 20° – 41° respectively with an average of 28.5° and 28.9° respectively. The LMTPJ plantar flexion also varied widely from 22° – 52°and 8° – 35° respectively with an average of 33.8° and 20.8° respectively (Table 3).

**Table 3:** Lesser metatarsophalangeal joint range of motion: pre and post implant

	Pre - implant						Post implant						
specimen	1	2	3	4	Mean (SD)	95%CI	1	2	3	4	Mean (SD)	95%CI	*p*-value
DORSIFLEXION (degrees)	12,34	12,65	52,5	31,6	33.78 (12.74)	13.51–54.05	21,89	20,15	32,48	40,95	19.84 (11.78)	0.78–38.91	0.1619
PLANTAR FLEXION (degrees)	28,66	22,02	32,75	51,75	27.27 (19.08)	3.09–57.63	12,32	28,45	8,1	34,5	26.62 (12.52)	6.70–38.91	0.9561

Table 3 shows that there was no significant difference in the average range of motion pre- and post- implant (note that a larger sample size could provide more clarity).

### Clinical stability

Stability was divided into stable, lax and dislocatable. Besides the one pre-implant specimen which was lax, all the others were stable both pre- and post-implant. The lax pre implant joint most probably stabilized with the soft tissue balance achieved with the implant (size of meniscus) (Table 4).

**Table 4:** Lesser metatarsophalangeal joint stability clinically: pre and post implant

	Pre-implant			Post-implant		
	Stable	Lax	Dislocatable	Stable	Lax	Dislocatable
1	•			•		
2	•			•		
3		•		•		
4	•			•		

### Stability with 5 kgf (49 N)

All the specimens both pre- and post-implant were stable in dorsal displacement and dorsiflexion using a 5 kg weight (49 N) (Table 5).

**Table 5:** Lesser metatarsophalangeal joint stability with 5 kgf (49 N) pre and post implant

	Pre-implant						Post-implant					
	Dorsal displacement			Dorsiflexion			Dorsal displacement			Dorsiflexion		
	Stable	Lax	Dislocatable	Stable	Lax	Dislocatable	Stable	Lax	Dislocatable	Stable	Lax	Dislocatable
1	•			•			•			•		
2	•			•			•			•		
3	•			•			•			•		
4	•			•			•			•		

## Discussion

Lesser metatarsophalangeal joint arthritis (primarily Freiberg’s infraction and post traumatic), may require surgical intervention once conservative management fails. There is poor evidence in supporting resection arthroplasty, excision interpositional arthroplasty, autografts and allografts [13–15]. To date there is no effective long-term replacement arthroplasty option. Silicone [17–26], ceramic [30] and metal LMTPJ arthroplasty [27, 28] have been reported with mixed success.

The use of silicone is associated with numerous complications [3, 34] including prosthetic loosening with failure, transfer lesions, local bone erosion, joint synovitis, infection secondary to impaired vascularity, lack of toe purchase with functional disability of the involved toe and foreign body reaction. As a result of the above problems, other materials such as titanium were introduced. Shih et al. described a case study using a titanium hemi-implant of the proximal phalanx. The benefit of this hemi-implant is that it does not alter the metatarsal parabola and allows for other surgical procedures to be performed in the future [28].

Total ceramic arthroplasty has been reported by Townshend and Greiss for painful, destructive disorders of the lesser MTPJs. All nine patients were female with a mean age of 51 years. The indications included primary and revision procedures. One case required a custom implant. Mean follow-up was 23 months with a mean AOFAS score of 75. Eight patients reported good or excellent results [30]. This was a small cohort with short follow-up.

Studies currently available are between case series and reports at level IV and V. The findings cannot be generalized or interpreted due to the low numbers, the retrospective nature of the studies and due to the rarity of the disease.

The idea of a total LMTPJ replacement arthroplasty remains a feasible option for the isolated arthritic LMTPJ. Although not subjected to large axial loads, these replacements still need to adhere to the basic principles of replacement implants and good soft tissue tension restoration to be successful. The available body of evidence around LMTPJ replacement arthroplasty comprises few studies of very small patient cohorts, and as such no grade of recommendation for any particular procedure or implant can be made with confidence [35].

For these reasons the author developed this LMTPJ replacement. The materials used in this implant (titanium and UHMWPE) are accepted internationally and the titanium nitride is proven to enhance surface hardness. The sizes of the metallic components (metatarsal and phalangeal) were determined by the accurate measurement of the respective bones on skeleton specimens. The metatarsal component fixation mechanism is unique. It has a “spring” intra medullary fixation mechanism with added “barbs” to increase the surface area. The mobile bearing is likewise unique in its attachment to the phalangeal component in that it is a completely rotating platform which allows a certain amount of multidirectional gliding and a wide range of motion. The phalangeal fixation is of the screw in type.

The implant is not a substitute for a poorly functioning or unbalanced ray in the forefoot. Large contact area is achieved between the component and the subchondral bone by virtue of the flat resection of the bone and the flat surface of the component.

The implant alone is by no means the stabilizing factor. Soft tissue balancing and fibrous tissue surrounding the implant provide the majority of strength to support the joint.

Cyclical loading of 5,000,000 cycles within physiological forces showed minimal wear of both the metatarsal implant and polyethylene meniscus.

The in vitro cadaveric studies allowed the researchers to develop and perfect the surgical technique, record the range of motion, determine the stability both clinically and by means of applied forces. The authors noted the large discrepancies in the range of motion pre and post- implant in some of the specimens and this was attributed to the quality of tissue in the cadaver. Nevertheless, the range of motion was maintained and even slightly improved in some of the specimens. The stability was excellent in both dorsal displacement and dorsiflexion.

There are few cadaveric studies pertaining to general loading and forces on the LMTPJ and there are no available cadaveric studies for LMTPJ replacement arthroplasty. Often implants in the development phase lack cadaveric trials and are only subjected to cyclical loading followed by clinical trials.

The relatively large bearing surfaces between the components will hopefully contribute to the longevity of the replacement arthroplasty but this will remain to be seen. In the event of implant failure with no possibility of a revision, the implant can be removed and the joint left as an “excision arthroplasty” which although not ideal has been described as a surgical option for Freiberg’s infraction or degenerative joint disease.

Considering published articles regarding cadaver utilization, this research presents an in vitro study which utilized 15 cadavers, culminating in six cadavers in the final stages (four for technique and measurement testing and two for assessment of surgical technique and X-rays by an independent foot and ankle surgeon).

## Conclusion

This novel LMTPJ replacement arthroplasty has been developed to fill the void of replacement arthroplasty options in the isolated arthritic LMTPJ. This novel three-component implant has high conformance and a large bearing surface. Cyclic loading of the implants under physiological loads has shown no signs of wear or damage.

This proof of concept study has shown this LMTPJ replacement to be simple in its surgical technique requiring minimal specialized instrumentation, achieving good range of motion and stability, albeit the inferior quality of cadaveric tissue, with good surgical reproducibility. This study is the basis for clinical trials (the implant has been cleared for clinical trials by Human Ethics of the University). For clinical trials, as with other replacements, the “ideal” candidate must be sought, followed by the stringent principles of replacements and informed consent.

## Data Availability

Please contact author for data requests.

## Abbreviations

LMTPJ: Lesser metatarsophalangeal joint
MTPJ: Metatarsophalangeal joint
UHMWPE: Ultra-high molecular weight polyethylene
